# Precise Pressure Control for Screw Extrusion 3D Printing of PP-GF Composites Based on Inverse Model Feedforward and Variable Structure Feedback

**DOI:** 10.3390/ma19071453

**Published:** 2026-04-05

**Authors:** Yunlong Ma, Xiping Li, Nan Ma, Youqiang Yao, Sisi Wang, Zhonglue Hu

**Affiliations:** 1 College of Engineering, Zhejiang Normal University, Jinhua 321004, China; mayunlong7717@163.com (Y.M.); lxp2010@zjnu.cn (X.L.); yaoyouqiang@zjnu.edu.cn (Y.Y.); sisi.wang@zjnu.edu.cn (S.W.); 2 Tianjin Institute of Aerospace Mechanical and Electrical Equipment, Tianjin 300456, China; manantianjin@163.com

**Keywords:** screw extrusion additive manufacturing, precise pressure control, smith predictor, variable structure feedback, interlayer bonding strength

## Abstract

Addressing challenges such as the non-Newtonian fluid characteristics of melt, significant system hysteresis, and rheological thermal drift in large-scale glass fiber-reinforced polypropylene (PP-GF) screw-extrusion additive manufacturing (SEAM), this paper proposes a composite pressure control strategy based on inverse model feedforward and variable-structure feedback (VSFC-Smith). This strategy establishes a dynamic pressure benchmark through an inverse rheological model, utilizes a Smith predictor to compensate for time delay, and introduces dead-zone variable-structure feedback to smoothly suppress thermal drift. Experimental results demonstrate that, compared to traditional PID (Proportional-Integral-Derivative) controller, the VSFC-Smith strategy reduces the step pressure overshoot from 23.37% to 17.37%, decreases steady-state screw speed fluctuation by approximately 50%, and limits the error within ±0.04 MPa during complex trajectory tracking. In practical molding validation, this strategy effectively suppressed surface ripples, reducing the surface roughness (Sa) by 14.5% to 124.41 μm; simultaneously, the Z-directional interlayer tensile strength reached 12.63 MPa (a 22.5% improvement compared to open-loop control). This study successfully overcomes the limitations of traditional high-gain feedback, achieving synergistic optimization of the macroscopic morphology and microscopic mechanical properties of composite parts.

## 1. Introduction

Screw extrusion additive manufacturing (SEAM), also known as fused granular fabrication (FGF), has emerged as a key technology for manufacturing large-scale composite components due to its high deposition rate (>5 kg/h) and the ability to directly utilize low-cost industrial granular feedstocks [1,2]. Among numerous engineering thermoplastics, glass fiber-reinforced polypropylene (PP-GF) exhibits immense application potential in the automotive and aerospace sectors, owing to its excellent specific modulus, chemical resistance, and cost-effectiveness [3,4,5]. However, while polymer melts in mature fused filament fabrication (FFF) inherently exhibit non-Newtonian behaviors, the highly filled semi-crystalline composites such as PP-GF demonstrate much more pronounced shear-thinning characteristics and complex rheological dependencies during FGF, posing significant challenges to existing control architectures [6,7].

Currently, mainstream commercial 3D printing firmware (e.g., Klipper and Marlin) is primarily designed for FFF systems. Their core control logic is based on linear kinematic assumptions, employing a “linear pressure advance” algorithm to compensate for extrusion lag, and presuming a strictly linear relationship between extrusion flow rate and feed motor speed [8]. Although unfilled PLA exhibits shear-thinning behavior at typical FFF shear rates (e.g., 0.1 to 100 s^−1^), linear control models remain viable. This is because the FFF extrusion process can be approximated as a mechanical piston model, where the feeding of the solid filament drives the melt, maintaining a proportional relationship between the feed rate and the extruded volume. In contrast, FGF relies on continuous screw extrusion, where such simplified models present a mechanistic mismatch. Specifically, compared to unfilled PLA, the addition of 28 wt% glass fibers in PP-GF elevates the overall melt viscosity, introduces yield stress, and reduces the flow behavior index (n). Consequently, the PP-GF melt demonstrates non-linear characteristics, rendering traditional linear control approaches inadequate for FGF systems. [9]. Rheological mechanisms indicate that changes in screw speed lead to a power-law decrease in apparent viscosity accompanied by shear-induced elastic stresses, causing a nonlinear surge in flow rate [10,11]. Consequently, adopting the open-loop control strategies from traditional FFF firmware results in extrusion volume prediction deviations during dynamic speed changes [12], leading to poor surface quality and high roughness in printed parts.

In-depth mechanistic analysis reveals that the precise control of PP-GF screw extrusion is constrained by three inherent physical limitations. Firstly, there is significant transport lag. The melt undergoes complex transport in the metering section of a screw with a large length-to-diameter ratio [13,14], leading to a pure time delay between the control input and the nozzle pressure response. This characteristic is prone to causing reduced control precision and instability in closed-loop systems, requiring complex distributed parameter models for stability analysis [15,16]. Secondly, there is strong nonlinear gain. The flow of high-viscosity non-Newtonian fluids in the mixing section of the screw is governed by power-law constitutive equations, exhibiting strong shear-thinning behavior [17]. The system process gain fluctuates drastically with shear rate and is influenced by the complex interplay of three-dimensional screw channel curvature and viscous dissipation [18], making it difficult for single-parameter controllers to maintain optimal performance across the entire speed range. Finally, there is time-varying rheological drift. During long-period printing, shear heating and conjugate heat transfer effects lead to changes in the melt’s thermal history [19], leading to slow pressure fluctuations in extrusion pressure at a constant screw speed, manifesting as a slow, time-varying disturbance that is difficult to model. These inherent physical limitations collectively result in unstable extrusion pressure, which is a cause of poor surface quality in open-loop printing.

Traditional PID solutions struggle to balance fast response and steady-state stability in SEAM systems facing significant time delays and random disturbances [20,21]. Aggressive PID parameter tuning, while capable of reducing rise time, often leads to severe overshoot and high-frequency oscillations in the actuator [22,23]. Such chatter not only exacerbates motor wear but also causes surface ripples in the extrudate, severely affecting product dimensional accuracy [24]. Crucially, even when PID control manages to stabilize the extrusion pressure, its aggressive response often results in significant fluctuations in screw speed. These rapid changes in screw speed directly translate to frequent variations in extrusion flow rate, which, despite pressure stability, can paradoxically degrade surface quality rather than improve it. Furthermore, traditional PID logic lacks the ability to identify disturbance types [25]. Under feedback delay, long-period rheological thermal drift can easily be misjudged as a sudden fault, leading to erroneous aggressive screw speed compensation. This over-regulation, caused by lag, not only fails to improve melt quality but also results in over-extrusion defects, compromising part forming accuracy.

Therefore, in high-performance composite manufacturing, achieving superior surface quality is paramount. While stable extrusion pressure is critical, equally important is the smooth and consistent extrusion flow rate, as unstable flow, even with seemingly stable pressure, directly leads to surface defects. To address these challenges and enhance the macroscopic surface quality of PP-GF components, this study proposes a robust composite pressure control system, termed VSFC-Smith. This strategy integrates inverse model feedforward to decouple nonlinearities, utilizes a Smith predictor to compensate for large time delays, and designs a variable-structure feedback controller (VSFC) with a benchmark regression mechanism. This approach aims to effectively suppress disturbances and melt viscosity drift, ensuring pressure stability while significantly improving actuator operational smoothness for consistent flow. Through comparative experiments, this study validates the effectiveness of VSFC-Smith in avoiding over-regulation, with the ultimate goal of synergistically optimizing the macroscopic and microscopic properties of printed parts, focusing on enhanced surface quality through a superior balance between pressure stability and screw speed smoothness.

## 2. Materials and Methods

### 2.1. Materials

This study employed polypropylene–glass fiber (PP-GF) composite material as the experimental subject. The material was prepared by compounding polypropylene pellets (RP344P, manufactured by LyondellBasell, Houston, TX, USA) and glass fibers (960A, from China Jushi Group, Jiaxing, China), followed by granulation using a twin-screw extruder (SHJ-35A, Nanjing Giant Machinery Co., Ltd., Nanjing, China). The average mass fraction of glass fibers in the composite pellets was 28 wt%. Key rheological and thermophysical properties are detailed in Table 1.

### 2.2. Experimental Platform

**Figure 1:**
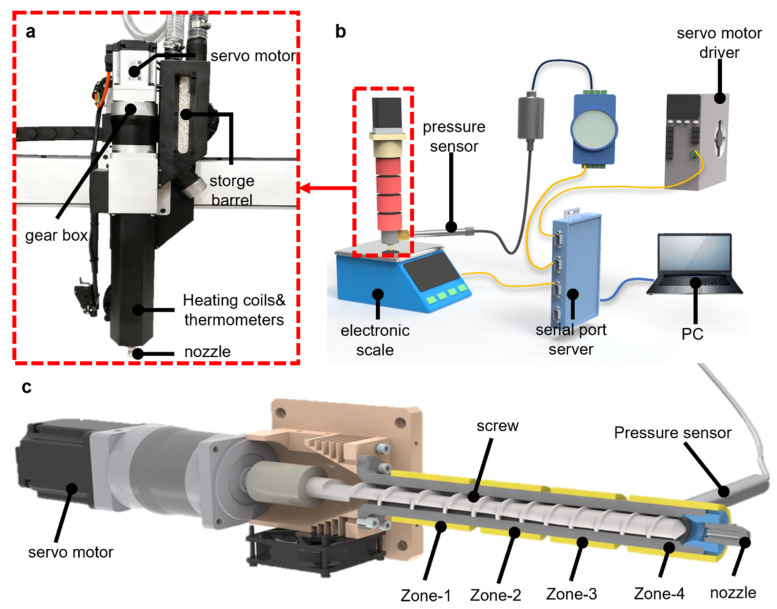
Overview of the large-scale screw extrusion-based additive manufacturing SEAM system. (**a**) The physical experimental platform; (**b**) The heterogeneous control and data acquisition architecture; (**c**) Cross-sectional view of the extrusion unit.

The experimental validation was performed on a large-scale SEAM system (modified CI Uni-Print MEGA), shown in Figure 1a, with a build volume of 1.5 × 1.5 × 1.5 m. The extrusion unit is driven by a high-torque servo motor. It features a four-zone ceramic heating system with embedded Pt1000 sensors, controlled by a zoned temperature strategy (Table 2) to maintain a consistent melt viscosity gradient. Due to the large thermal inertia of the heating elements, this temperature control loop operates on a much slower timescale than the motor-driven pressure control. A 3.0 mm hardened steel nozzle, with an independent heating unit, resists glass fiber abrasion and ensures smooth material flow.

As depicted in Figure 1b, this architecture decouples motion control and extrusion control at the physical level and constructs a synchronous data acquisition network. Klipper firmware controls the X/Y/Z axes, while the screw extrusion motor operates independently. Nozzle pressure signals are digitized via an analog module and transmitted via RS485, with all data precisely timestamp-aligned.

Figure 1c illustrates the extrusion mechanism’s cross-section, featuring a custom screw (20 mm diameter, 15:1 L/D ratio). This highlights a critical physical constraint: the actuator is at the screw’s rear, while the pressure sensor is in the front nozzle cavity. This significant spatial separation causes the melt pressure response to lag motor speed changes, introducing a non-negligible time delay (τ) into the control loop, thus necessitating the advanced predictive control strategy proposed in Section 3.

### 2.3. Control System Architecture and Data Flow

**Figure 2:**
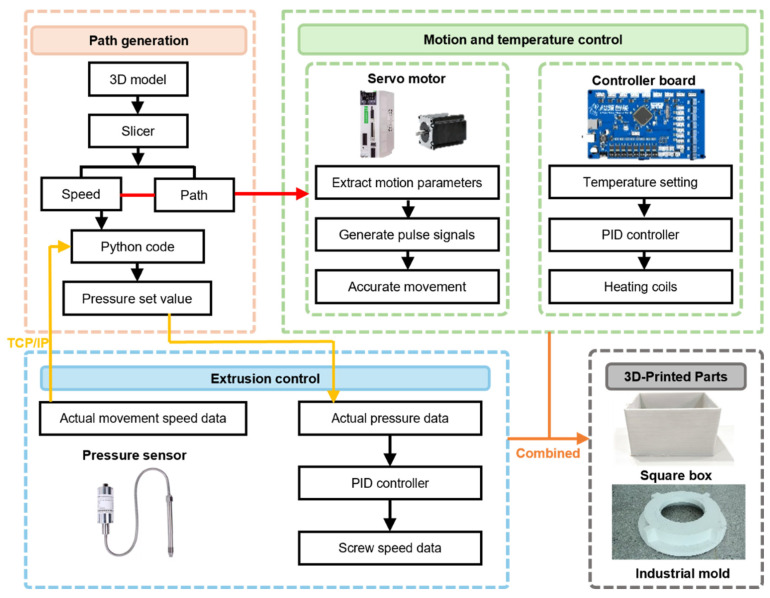
Architecture of screw extrusion control system based on pressure feedback.

To implement the proposed composite control strategy, a parallel and collaborative software architecture was developed, building upon the experimental platform detailed in Section 2.2. As depicted in Figure 2, the system employs a dual-channel parallel topology, physically decoupling high-precision geometric motion control from complex rheological extrusion regulation. This decoupling enables independent, high-frequency adjustment of extrusion flow by the host computer, forming the basis for the pressure closed-loop control.

The motion control subsystem (green dashed box in Figure 2) utilizes an underlying MCU (Microcontroller Unit) equipped with embedded firmware. It parses G-code, executes microsecond-level kinematic interpolation for X/Y/Z axes, and performs PID closed-loop control for heating, thereby ensuring real-time geometric trajectory accuracy. The extrusion control subsystem (blue area in Figure 2) operates on the host computer, executing the composite control strategy. A Python (version 3.11) supervisory program extracts G-code velocity vectors in parallel to calculate the inverse model feedforward quantity $\Omega_{ff}$ and determine the theoretical target pressure $P_{target}$ via a rheological model.

A 50 Hz soft real-time feedback loop is established via TCP/IP. To prevent network TCP/IP jitter from blocking control execution, the extrusion control subsystem runs on a host PC (AMD Ryzen 9 7945HX, 16GB RAM) using a dual-rate asynchronous multi-threading architecture. Sensor data acquisition and motor command transmission operate in background threads with a 50 Hz sampling period (20 ms interval). The main control thread retrieves state variables via non-blocking memory locks and executes the Kalman filter and VSFC-Smith algorithms at a 20 Hz control period (50 ms interval). Profiling over 10,000 cycles shows a maximum computational latency of 0.224 ms per cycle. Consuming less than 0.5% of the 50 ms time budget, this eliminates task starvation and ensures soft real-time control. The host computer issues control commands and records pressure, screw speed, and material weight data. Pressure sensor data are timestamp-synchronized with motion commands to eliminate communication delay errors. If pressure deviates, the controller, after Smith predictor processing, calculates a screw speed compensation vector $\Delta \Omega_{fb}\left(t\right)$ and superimposes it onto the basic motion command. This architecture allows for real-time execution of computationally intensive algorithms on the host computer, achieving dynamic feedforward compensation and precise closed-loop rheological state regulation.

## 3. Results and Discussion

### 3.1. Open-Loop Characterization and Dynamic Analysis

This study conducted steady-state calibration and dynamic excitation tests under open-loop conditions to elucidate the screw extrusion process mechanism and construct an accurate controlled object model. Analysis of flow rate and pressure response data revealed the system’s dual characteristics: linear transport capacity and complex nonlinear pressure dynamics.

**Figure 3:**
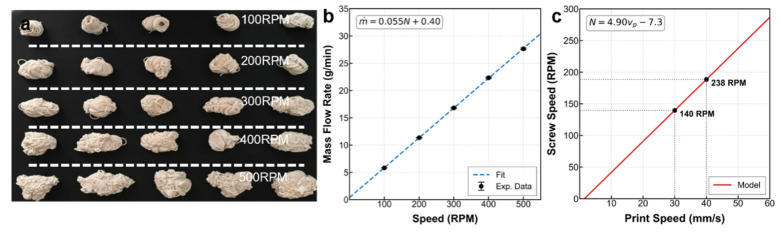
Steady-state characterization and modeling: (**a**) morphology; (**b**) mass flow rate vs. RPM; and (**c**) relationship between screw rotation speed and printing speed.

Firstly, establishing a deterministic mapping relationship between extrusion flow rate and screw speed is a prerequisite for implementing inverse model feedforward control. The theoretical volumetric flow rate $Q_{out}$ and screw speed N exhibit a geometric relationship based on the single-screw drag flow mechanism [26]:

(1) $$Q_{out}=\frac{1}{2}\pi^{2}D^{2}H_{3}Nsin\theta cos\theta$$

where D is the screw diameter, $H_{3}$ is the metering section channel depth, and θ is the helix angle. This equation reveals a theoretically strict linear relationship between flow rate and screw speed. To validate this model and establish the correlation between extrusion volume and print geometry, calibration tests were performed within the 100–500 RPM range. Figure 3a illustrates the macroscopic morphology of the extrudate at different screw speeds, with mass flow rates at each speed point obtained through timed weighing. Figure 3b presents the quantitative data derived from these measurements, showing that the actual mass flow rate $\dot{m}$ highly conforms to a linear equation with screw speed N ($R^{2}=\;0.998$), thereby establishing the system’s linear conveying baseline.

(2) $$A_{cs}=\left(W-H\right)H+\frac{\pi H^{2}}{4}$$

Here, W is the line width, and H is the layer height. This geometric constraint model provides a physical baseline for subsequently converting mass flow rate into equivalent deposited volumetric flow rate. Based on the calibrated linear model and the mass conservation constraint of Equation (2), the coupling relationship between extrusion supply and printing motion was derived. As shown in Figure 3c, the screw speed required to maintain specific line width and layer height exhibits a strict linear dependence on the printing movement speed. This clear linear mapping constitutes the static feedforward law within the control strategy in Section 3, enabling the controller to make real-time estimates and respond to the required basic screw speed based on path planning speed.

**Figure 4:**
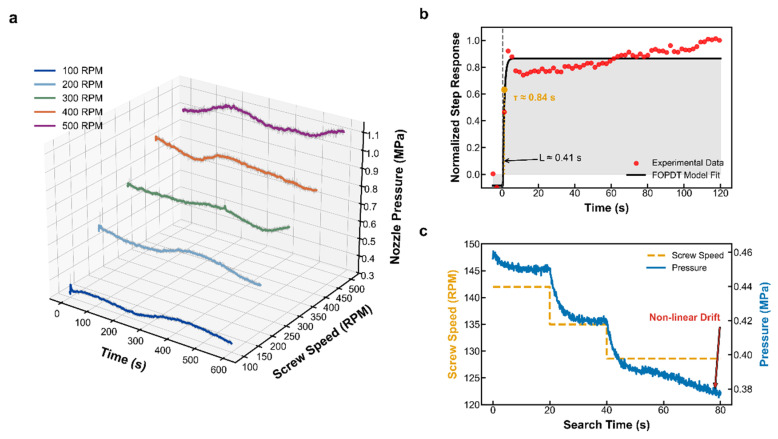
Experimental characterization of the nozzle pressure system: (**a**) steady-state noise analysis, (**b**) dynamic step response identification, and (**c**) operating point optimization process.

Figure 4 demonstrates that, in contrast to the linear flow rate output, internal nozzle pressure exhibits complex nonlinear dynamic features. Figure 4a, showing steady-state noise analysis, reveals that the raw pressure signal is consistently accompanied by random disturbances with a variance of $\sigma^{2}=4.98\times e^{-5}$. This background noise, caused by glass fiber rearrangement and local recirculation, does not disappear with increasing screw speed. This indicates that simple high-gain error feedback is highly susceptible to noise interference, leading to actuator oscillation, thus establishing the necessity of introducing a dead-zone control logic.

The step response curve in Figure 4b uncovers the system’s inherent physical inertia. FOPDT (First-Order Plus Dead Time) model fitting demonstrates a physical delay of L = 0.41 s from command issuance to pressure establishment, followed by an exponential rise process with τ = 0.84 s. This significant dynamic lag directly necessitates the design of a Smith predictor. Furthermore, as depicted in Figure 4c, the operating point pressure undergoes a slow rheological drift over time, influenced by shear heating and thermal history accumulation. These inherent nonlinear dynamic constraints, in sharp contrast to the excellent linear flow rate characteristics, collectively establish the physical design principles for the composite control strategy in the next section: namely, utilizing feedforward to ensure precise material deposition for improved surface quality, and incorporating a Smith predictor with dead-zone feedback to stabilize the extrusion state and minimize flow rate fluctuations.

### 3.2. Mathematical Model of Nozzle Internal Pressure

While Section 3.1 establishes the screw speed feedforward baseline, open-loop control fails to suppress pressure fluctuations from rheological disturbances, leading to unstable extrusion conditions and inconsistent material deposition, which directly impacts surface quality. Therefore, this section aims to calculate the steady-state target pressure ($P_{target}$) that ensures optimal and relatively stable material flow by establishing an inverse physical model, serving as a reference anchor for the closed-loop system to maintain a stable rheological state.

To derive this setpoint, a physical rheological model of the nozzle interior is essential. PP-GF melt flow in a circular nozzle follows non-Newtonian fluid dynamics, where volumetric flow rate Q and pressure drop ΔP satisfy the modified Hagen–Poiseuille law:

(3) $$Q={\left(\frac{\Delta P}{2L}\right)}^{\frac{1}{n}}\frac{\pi n}{\eta_{0}\left(3n+1\right)}R^{3+\frac{1}{n}}$$

**Figure 5:**
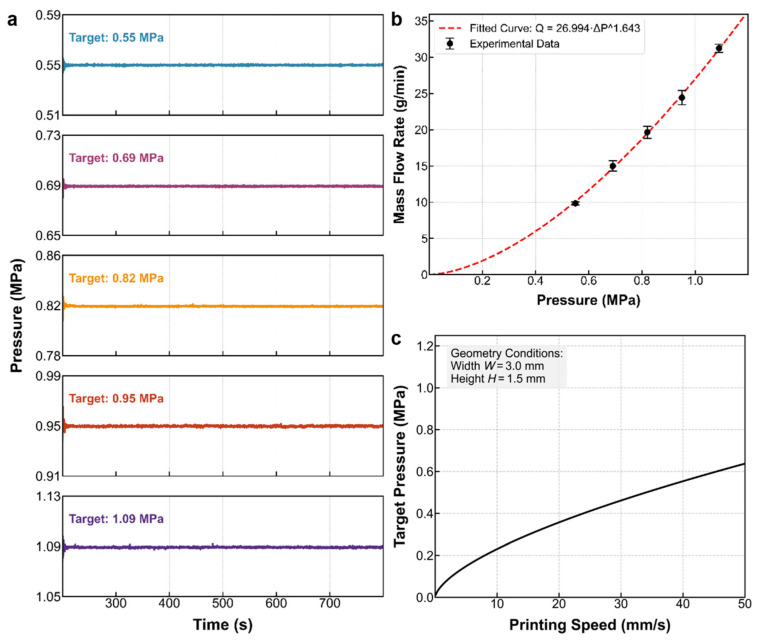
Steady-state extrusion characterization and feed rate control modeling. (**a**) Time-domain pressure response stabilized by PID control at discrete target levels. (**b**) Fitting of the constitutive model relating mass flow rate to nozzle pressure. (**c**) Feedforward control curve mapping target pressure to printing speed.

Here, R and L are the nozzle radius and flow channel length, respectively, while n and $\eta_{0}$ are the material’s rheological parameters. This equation highlights the nonlinear coupling between pressure and flow rate. For real-time control, the theoretical model is simplified into a power-law form, with parameters identified through steady-state experiments. As shown in Figure 5a, nozzle pressure was stabilized at discrete levels (0.55–1.09 MPa) using a PID controller, and corresponding mass flow rates were recorded. Regression analysis (Figure 5b) reveals a significant power-law relationship between mass flow rate $\dot{m}$ and pressure P:

(4) $$\dot{m}=k\cdot P^{\alpha }$$

The fitting results show k = 26.5 and a shear-thinning index α = 1.58. An α > 1 implies that absolute pressure locking would cause drastic screw speed adjustments and actuator oscillation. Therefore, the control strategy prioritizes constraining pressure fluctuations within a minimal range, maintaining a smooth screw speed while ensuring stable material deposition and improved surface quality.

Finally, to construct a reference baseline for closed-loop control, we inversely solve Equation (4) by substituting the geometric flow rate requirement established in Section 3.1 ($\dot{m}=\rho A_{cs}v_{print}$). Based on the principle of mass conservation, the target pressure control law corresponding to print speed $v_{print}$ can be derived:

(5) $$P_{target}={\left(\frac{\rho A_{cs}}{k}\cdot v_{print}\right)}^{\frac{1}{\alpha }}$$

Substituting the experimentally identified parameters and target geometric conditions (W = 3.0 mm, H = 1.5 mm), the resulting control curve is shown in Figure 5c. This curve defines the target pressure trajectory required to ensure flow matching and maintain stable extrusion at different print speeds. In the control strategy presented in this paper, the system will use this value as its target, maintaining screw speed feedforward tracking while using closed-loop fine-tuning to control pressure along this trajectory, thereby mitigating the adverse effects of open-loop fluctuations on extrusion stability and, consequently, surface quality.

### 3.3. Control Strategy Implementation

**Figure 6:**
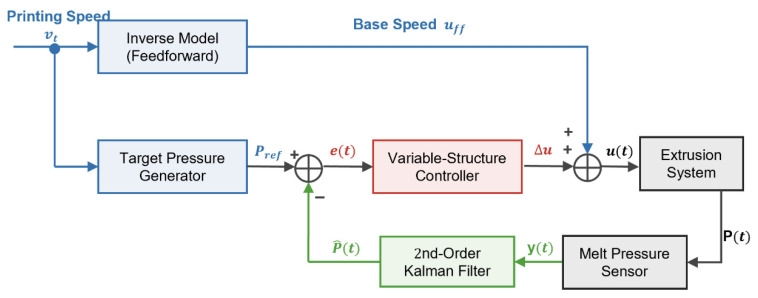
Block diagram of the composite control architecture integrating inverse model feedforward and variable-structure feedback.

To address challenges such as transport lag, nonlinear gain, and rheological thermal drift, this study proposes the VSFC-Smith composite control architecture for PP-GF screw extrusion. As shown in Figure 6, this system employs a dual-degree-of-freedom structure with feedforward and feedback. The feedforward path utilizes an inverse rheological model to decouple static nonlinearities, while the feedback path suppresses dynamic disturbances. The total control law $u\left(t\right)$ applied to the extrusion motor is defined as the linear superposition of the feedforward control quantity $\Omega_{ff}\left(t\right)$ and the feedback compensation quantity $\Delta \Omega_{fb}\left(t\right)$:

(6) $$u\left(t\right)=\Omega_{ff}\left(t\right)+\Delta \Omega_{fb}\left(t\right)$$

Here, the feedforward quantity $\Omega_{ff}\left(t\right)$ invokes the screw speed–print speed linear mapping model established in Section 3.1 (Figure 3c), determining the flow rate demand caused by changes in print speed and establishing the system’s open-loop response baseline. Simultaneously, the pressure reference generation path invokes the inverse constitutive equation derived in Section 3.2 (Equation (5)) to calculate in real time the corresponding target pressure trajectory $P_{target}$. This trajectory serves as the dynamic rheological anchor for the closed-loop control system, crucial for maintaining extrusion stability and improving surface quality. $\Delta \Omega_{fb}\left(t\right)$ is generated by a closed-loop controller integrating a Smith predictor and variable structure logic, used to compensate for lag and suppress rheological drift.

First, based on the dynamic analysis data from Section 3.1 the dynamic characteristics of the extrusion process are approximated as a first-order inertial plus pure delay (FOPDT) model:

(7) $$G_{p}\left(s\right)=\frac{K}{\tau s+1}e^{-Ls}$$

Here, K is the process gain, τ=0.84 s is the time constant, and L=0.41 s is the pure delay time. The Smith predictor removes the pure delay factor $e^{-Ls}$ from the characteristic equation by paralleling an estimation model $G_{m}\left(s\right)\left(1-e^{-Ls}\right)$ without the delay term in the feedback loop. The modified closed-loop transfer function is equivalent to:

(8) $$G_{closed}\left(s\right)=\frac{{C\left(s\right)G}_{p}^{*}\left(s\right)}{1+{C\left(s\right)G}_{p}^{*}\left(s\right)}e^{-Ls}$$

Here, $G_{p}^{*}\left(s\right)$ is the nominal model without delay. Through this transformation, the design of the feedback controller $C\left(s\right)$ is no longer limited by the delay term, thus allowing for larger gains to improve the system’s dynamic tracking performance.

To suppress the time-varying drift of melt viscosity caused by thermal accumulation during long-period printing, and to prevent the integrator from over-accumulating and over-regulating this slow, benign drift, this study adopts a variable structure feedback controller (VSFC) with dead-zone logic. This strategy dynamically adjusts the integral gain based on the magnitude of the pressure error $e\left(t\right)$, with its feedback control law being:

(9) $$\Delta \Omega_{fb}\left(t\right)=K_{p}e\left(t\right)+K_{i}\left(e\right)\int_{0}^{t}e\left(\tau \right)d\tau$$

Here, the integral gain $K_{i\left(e\right)}$ is a piecewise function that varies with the error state, with the specific logic as follows:

(10) $$K_{i\left(e\right)}=\left\{\begin{array}{cc} 0 & \left|e\right|<\delta \\ \alpha \cdot K_{i,base} & \delta \leq \left|e\right|<\xi \\ K_{i,base} & \left|e\right|\geq \xi \end{array}\right.$$

This logic divides the control domain into three functional regions. When pressure fluctuations are within the preset quality tunnel ($\left|e\right|<\delta$), the controller shields small errors from accumulating in the integral term and introduces a baseline regression mechanism, driving the screw speed to gradually converge back to the feedforward nominal operating condition $\Omega_{ff}\left(t\right)$. When the error magnitude slightly increases ($\delta \leq \left|e\right|<\xi$), the system smoothly transitions to the creep zone, enabling low-gain integral action to eliminate steady-state deviations. Only when a significant pressure deviation ($\left|e\right|\geq \xi$) is detected does the controller restore full gain to achieve rapid pressure reconstruction. The dead-zone thresholds (δ, ξ) are systematically optimized using the steady-state pressure fluctuations of the 28 wt% PP-GF melt. Specifically, the baseline pressure fluctuation component is isolated to calculate its standard deviation (σ), which adaptively defines the boundaries (e.g., utilizing the 3σ rule). This approach offers a highly statistical methodology that can be readily extended to other polymer melts. Through this variable structure strategy, the system achieves an optimal balance between surface quality and equipment operating stability while suppressing microscopic thermal drift. Regarding the potential coupling effects between the multi-zone temperature control (Table 2) and the pressure control, as noted in Section 2.2, the two loops operate on different timescales. Due to this timescale separation, temperature fluctuations and long-term heat accumulation act as slow, time-varying disturbances to the pressure loop. The variable-structure integral logic within the VSFC-Smith strategy treats these thermal variations as general baseline drifts, dynamically suppressing them to maintain pressure stability without requiring an additional multi-variable decoupling controller.

### 3.4. Control System Performance Evaluation

**Figure 7:**
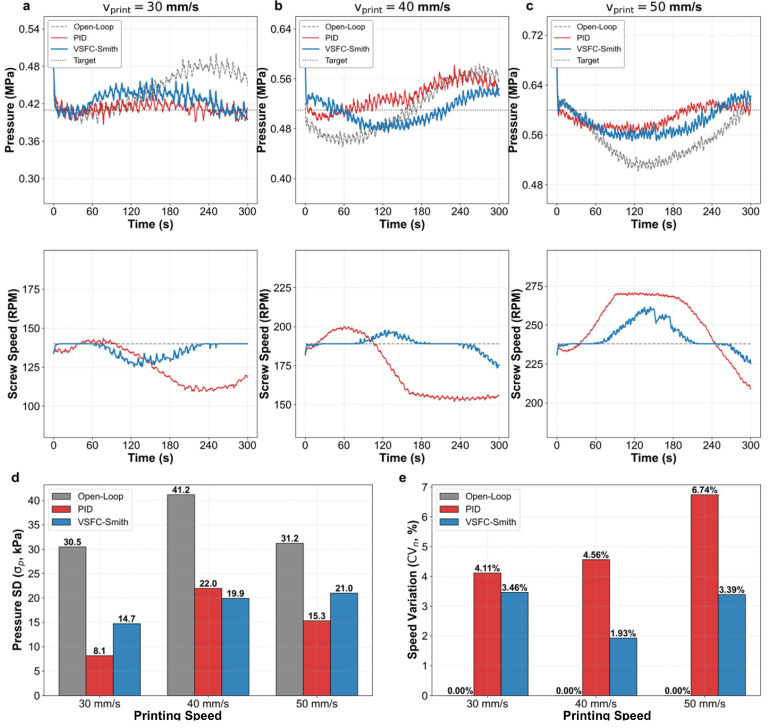
Experimental comparison of steady-state and dynamic pressure regulation performance under varying printing speeds. (**a**–**c**) Steady-state pressure time-domain responses and corresponding screw speed curves for open-loop, PID, and VSFC-Smith control at printing speeds of 30 mm/s, 40 mm/s, and 50 mm/s, respectively. The upper panels show pressure (MPa) over time (s), and the lower panels show screw speed (RPM) over time (s). (**d**) Quantitative comparison of pressure standard deviation ($\sigma_{p}$) for different control strategies across varying printing speeds. (**e**) Quantitative comparison of screw speed fluctuation rate (${CV}_{n}$) for different control strategies across varying printing speeds.

This paper evaluates the dynamic control performance of the VSFC-Smith strategy through constant-speed extrusion, step response, and sinusoidal trajectory tracking experiments. Melt pressure stability is a critical physical prerequisite for ensuring uniform extrusion and high-quality surface formation of PP-GF composites. The upper panels of Figure 7a–c display the steady-state pressure time-domain responses at different printing speeds. Open-loop control, unable to compensate for rheological thermal drift, resulted in significant pressure baseline shifts and severe fluctuations. After introducing closed-loop control, both PID and VSFC-Smith effectively constrained the pressure near the target value. However, the screw speed curves in the lower panels of Figure 7a–c intuitively show that PID control exhibits significant screw speed fluctuations, whereas the VSFC-Smith strategy demonstrates markedly smaller speed fluctuations.

Quantitative data (Figure 7d) indicate that PID control achieves the lowest pressure standard deviation (e.g., $\sigma_{p}$ of only 8.1 kPa at 30 mm/s). However, this minimal steady-state error comes at the cost of high-frequency actuator oscillation. To suppress rheological background noise, PID causes severe screw speed fluctuations, with its coefficient of variation (${CV}_{n}$) reaching as high as 6.74% at 50 mm/s (Figure 7e). This not only exacerbates mechanical wear but also induces periodic pulsations in the macroscopic extrusion line width, severely damaging the surface quality of the manufactured parts. In contrast, the VSFC-Smith strategy, while maintaining comparable pressure stability ($\sigma_{p}$ ranging from 14.7 to 21.0 kPa), filtered high-frequency rheological noise through the dead-zone logic in its variable structure feedback. This mechanism reduced the screw speed fluctuation rate (${CV}_{n}$) by approximately 50%, significantly improving equipment operational smoothness and lifespan while satisfying the constant pressure conditions required for stable and uniform material deposition.

**Figure 8:**
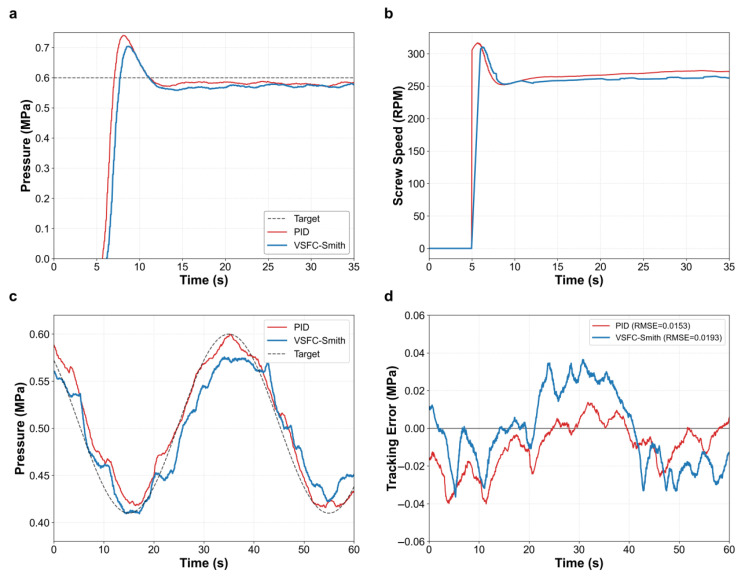
Experimental evaluation of dynamic pressure tracking performance. (**a**) Step response of nozzle pressure. (**b**) Corresponding control input (screw rotation speed). (**c**) Sine wave trajectory tracking performance. (**d**) Dynamic tracking error. The red and blue lines represent PID and VSFC-Smith, respectively.

The step response test from 0 to 0.6 MPa, shown in Figure 8a, further validates the system’s transient control capability, which is crucial for the initial path formation and surface quality of printed parts. Limited by the inherent pure time delay of the extrusion system, the high-gain feedback of PID control leads to a severe pressure overshoot of 23.37% (peak exceeding 0.74 MPa), which can easily result in excessive material accumulation during the initial stage. Combined with the control input in Figure 8b, it is evident that the screw speed command for PID exhibited a sharp surge at the moment of initiation, whereas the screw speed ramp-up for VSFC-Smith was significantly smoother. While maintaining similar rise times (1.06 s versus 0.96 s), VSFC-Smith successfully reduced the pressure overshoot to 17.37%. Furthermore, VSFC-Smith smoothly eliminated residual errors through a steady-state fine-tuning mechanism, avoiding the high-frequency oscillations characteristic of PID, thereby fundamentally improving start-up defects.

The sinusoidal trajectory tracking experiment (Figure 8c) simulated complex operating conditions with frequent acceleration and deceleration. Error statistics (Figure 8d) indicate that the Root Mean Square Error (RMSE) of VSFC-Smith (0.0193 MPa) was slightly higher than that of PID (0.0153 MPa). However, this absolute difference of approximately 0.004 MPa has a negligible physical impact on the rheological state. When processing highly shear-thinning melts, PID overreacted to high-frequency noise to maintain minimal error, leading to high-frequency pulsations in the control output. In contrast, VSFC-Smith utilized the synergistic effect of inverse model feedforward and dead-zone logic to actively filter out ineffective high-frequency disturbances. This strategy not only strictly limited the dynamic tracking error within ±0.04 MPa but also ensured smooth and coherent screw speed commands. Under continuous variable-speed conditions, VSFC-Smith demonstrated comprehensive control capabilities characterized by high smoothness and low overshoot, providing an exceptionally uniform extrusion and deposition environment, beneficial for surface quality of large-sized composite parts.

### 3.5. Impact of Pressure Fluctuations on Printing Quality and Control Validation

**Figure 9:**
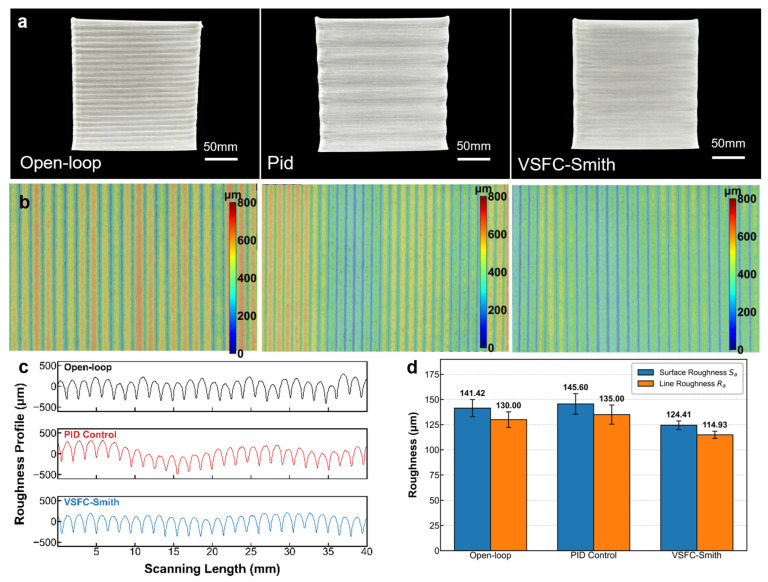
Comparison of surface quality of PP-GF parts under different nozzle pressure control strategies. (**a**) Macroscopic photographs of specimens printed with Open-loop, PID, and VSFC-Smith control; (**b**) Surface height color maps obtained by ultra-depth-of-field microscopy; (**c**) Cross-sectional roughness profiles along the scanning path (after detrending); (**d**) Comparative analysis of surface roughness (Sa) and line roughness (Ra) for different control strategies.

To comprehensively validate the practical control efficacy of the VSFC-Smith algorithm in screw extrusion-based 3D printing of PP-GF composites, this study systematically compared specimens fabricated under open-loop, PID, and VSFC-Smith control strategies across surface morphology (Figure 9).

The surface quality of the fabricated parts directly reflects the control algorithm’s ability to balance extrusion stability and pressure control. Figure 9a illustrates that open-loop controlled specimens exhibited irregular lines on their sidewalls, presenting a “stair-stepping effect”. While the introduction of PID control eliminated abrupt defects, it induced significant wavy patterns. This phenomenon stems from the inherent lag in the extrusion system, where PID, in an attempt to suppress pressure deviations, forced frequent oscillatory adjustments of the screw speed, leading to dynamic pulsations in the extruded line width. The ultra-depth-of-field 3D height cloud map in Figure 9b and the measurement data in Figure 9c confirm that under PID control, the surface roughness (Sa) paradoxically increased to 145.60 μm, which was higher than that of open-loop control (141.42 μm). This indicates that blindly introducing closed-loop feedback without effective compensation for time lag, while improving steady-state accuracy, sacrifices surface smoothness. In contrast, specimens produced with the VSFC-Smith strategy demonstrated optimal surface quality. Benefiting from the effective compensation of system time lag by the Smith predictor, this algorithm maintained stable pressure while preventing excessive oscillation of the control variable. Measurement results show that under the VSFC-Smith strategy, the surface roughness significantly decreased to 124.41 μm (approximately 14.5% lower than PID), and the line roughness (Ra) also dropped to a minimum of 114.93 μm, successfully resolving the ripple issue caused by PID and achieving a smooth surface.

To further quantify the improvements in surface quality, Figure 9d presents a comparative analysis of the surface roughness (Sa) and line roughness (Ra) for specimens produced under different control strategies. As indicated by the newly added error bars, the VSFC-Smith strategy not only reduces the mean roughness values but also narrows the measurement variance (e.g., the standard deviation of Sa drops from 10.2 μm under PID to 4.3 μm), demonstrating improved process repeatability. Statistical analysis confirms that the reductions in both Sa and Ra under VSFC-Smith control exhibit greater consistency across multiple print batches, with reduced dispersion. The concurrent decrease in both absolute roughness values and their dispersion directly validates the effectiveness of the VSFC-Smith strategy in achieving a more uniform macroscopic morphology.

It should be noted that the experimental validation in this study is currently limited to a single composite formulation, specifically PP-GF with a 28 wt% glass fiber mass fraction. Because varying the glass fiber concentration significantly alters the overall melt viscosity, introduces yield stress, and changes the flow behavior index, the specific mathematical parameters of the inverse rheological model derived in this work cannot be universally applied to all PP-GF composites. Consequently, a methodological limitation is that applying this control strategy to materials with different fiber concentrations or entirely different polymer matrices requires re-calibrating the inverse physical model through steady-state experiments to capture the specific non-Newtonian flow behaviors. However, while the specific power-law parameters are material-dependent, the overarching VSFC-Smith methodology is designed to be highly adaptable. By utilizing a Smith predictor to compensate for transport lag and variable-structure feedback to suppress rheological drift, the fundamental control architecture remains applicable to a broad range of highly filled, shear-thinning composite melts once the initial model calibration is performed.

## 4. Conclusions

This paper addresses the challenges of nonlinear gain, significant transmission lag, and long-period rheological thermal drift in large-scale PP-GF screw extrusion additive manufacturing. It proposes and validates a precision pressure composite control strategy based on inverse model feedforward and variable structure feedback (VSFC-Smith). The research demonstrates that traditional PID control, constrained by high-gain feedback, is prone to inducing high-frequency actuator oscillations and severe transient pressure overshoot when dealing with large time delays and rheological noise, thereby deteriorating the surface quality of fabricated parts. The VSFC-Smith strategy effectively compensates for system lag through a Smith predictor, significantly suppressing over-extrusion during the initial stage. Concurrently, its dead-zone tolerance mechanism successfully filters out high-frequency ineffective disturbances, achieving highly smooth dynamic pressure tracking under continuous variable-speed operating conditions.

Practical forming validation shows that this control architecture successfully achieves synergistic optimization of macroscopic geometric accuracy and microscopic interlayer mechanical properties. It effectively ameliorates the sidewall ripple defects caused by PID control, substantially enhances surface flatness, and maintains excellent Z-direction interlayer tensile strength while ensuring a stable extrusion flow field. This study overcomes the limitations of traditional high-gain feedback in non-Newtonian fluid extrusion, confirming the engineering effectiveness of “trading moderate transient errors for global flow field stability”, and provides a highly robust process control paradigm for the additive manufacturing of shear-thinning composite materials.

## Figures and Tables

**Table 1:** Physical Properties of PP-GF Composite Material.

Parameters	Standards	Data
Density	ISO 1183	1.11 $\;g/{cm}^{3}$
Melt Flow Rate (230 °C with 2.16 kg load)	ISO 1133	4.8 g/10 min
Heat Deflection Temperature	ISO 75-1.2	80 °C
Young’s modulus	ISO 527,1A	1129 MPa
Tensile Strength	ISO 527,1A	33.4 MPa
Bending strength	ISO 14125	51.1 MPa

**Table 2:** The temperature settings for each segment for printing the PP-GF.

Segments	Zone-1	Zone-2	Zone-3	Zone-4	Nozzle
Temperatures (°C)	120	180	180	190	190

## Data Availability

The original contributions presented in this study are included in the article. Further inquiries can be directed to the corresponding author.
